# Increased Expression of the Large Conductance, Calcium-Activated K^+^ (BK) Channel in Adult-Onset Neuronal Ceroid Lipofuscinosis

**DOI:** 10.1371/journal.pone.0125205

**Published:** 2015-04-23

**Authors:** Julien Donnelier, Samuel T. Braun, Natalia Dolzhanskaya, Eva Ahrendt, Andrew P. Braun, Milen Velinov, Janice E. A. Braun

**Affiliations:** 1 Department of Biochemistry and Molecular Biology, Hotchkiss Brain Institute, Cumming School of Medicine, University of Calgary, Calgary, Alberta, Canada; 2 Department of Physiology and Pharmacology, Hotchkiss Brain Institute, Cumming School of Medicine, University of Calgary, Calgary, Alberta, Canada; 3 Albert Einstein College of Medicine, Bronx, New York, United States of America; 4 New York State Institute for Basic Research in Developmental Disabilities, Staten Island, New York, United States of America; University of Houston, UNITED STATES

## Abstract

Cysteine string protein (CSPα) is a presynaptic J protein co-chaperone that opposes neurodegeneration. Mutations in CSPα (i.e., Leu115 to Arg substitution or deletion (Δ) of Leu116) cause adult neuronal ceroid lipofuscinosis (ANCL), a dominantly inherited neurodegenerative disease. We have previously demonstrated that CSPα limits the expression of large conductance, calcium-activated K^+^ (BK) channels in neurons, which may impact synaptic excitability and neurotransmission. Here we show by western blot analysis that expression of the pore-forming BKα subunit is elevated ~2.5 fold in the post-mortem cortex of a 36-year-old patient with the Leu116∆ CSPα mutation. Moreover, we find that the increase in BKα subunit level is selective for ANCL and not a general feature of neurodegenerative conditions. While reduced levels of CSPα are found in some postmortem cortex specimens from Alzheimer’s disease patients, we find no concomitant increase in BKα subunit expression in Alzheimer’s specimens. Both CSPα monomer and oligomer expression are reduced in synaptosomes prepared from ANCL cortex compared with control. In a cultured neuronal cell model, CSPα oligomers are short lived. The results of this study indicate that the Leu116∆ mutation leads to elevated BKα subunit levels in human cortex and extend our initial work in rodent models demonstrating the modulation of BKα subunit levels by the same CSPα mutation. While the precise sequence of pathogenic events still remains to be elucidated, our findings suggest that dysregulation of BK channels may contribute to neurodegeneration in ANCL.

## Introduction

Cysteine string protein (CSPα) is a synaptic vesicle protein and molecular chaperone that is essential for neuroprotection. Mutations in CSPα, L115R and L116Δ, cause adult neuronal ceroid lipofuscinosis (ANCL), a neurodegenerative disease characterized by the lysosomal accumulation of auto-fluorescent storage material, lipofuscin [1–3]. CSPα is comprised of an N terminal “J domain”, a hydrophobic stretch of residues followed by the characteristic cysteine string region and a C terminal domain thought to bind client proteins [4]. The mutations L115R and L116Δ that cause ANCL are in the cysteine string region and disrupt anchoring of CSPα to synaptic vesicles [5], most likely leading to a loss-of-chaperone-function at the synaptic vesicle and a toxic gain-of-function of mis-localized CSPα.

The role of CSPα-mediated synapse protection in neurodegenerative diseases remains a central biological question. Recognition of the importance of CSPα in the defense against neurodegeneration has fueled the pursuit of strategies to reinforce CSPα’s neuroprotective activity. CSPα KO mice exhibit fulminant neurodegeneration that is activity-dependent and have a shortened lifespan [6,7]. In *Drosophila*, CSPα KO’s are characterized by uncoordinated movements, shaking, temperature-sensitive paralysis and reduced lifespan [8]. In *C elegans*, CSPα null mutants show age-dependent sensorimotor defects, neurodegeneration and reduced lifespan [9]. Understanding the biochemical sequence of events underlying CSPα-mediated neuroprotection is critical in order to evaluate the efficacy and safety of therapeutics targeting CSPα. The assembly of CSPα with Hsc70 (heat shock cognate protein of 70kDa) and SGT (small glutamine rich tetratricopeptide repeat protein) to prevent synapse loss is an important feature of current models of the biochemical pathway underlying CSPα-mediated-neuroprotection [6,10–12]. As chaperone systems, in general, are responsible for the dynamic balance between promoting protein folding and directing proteins to degradation via the quality control machineries, the conformational work performed by the CSPα/Hsc70/SGT complex is likely important for maintaining the functional integrity of presynaptic protein clients.

We have recently reported that the expression of large conductance, calcium-activated K^+^ (BK) channels at the cell surface is regulated by CSPα [13,14]. BK channels are activated by both membrane depolarization and elevated intracellular Ca^2+^ levels and are central to neuronal excitability and neurotransmitter release. BK channel activity is regulated by a number of pre- and post-translational events and several conditions are further reported to influence channel expression at the plasma membrane, such as auxiliary BK β subunits, alternative splicing of the pore-forming α subunit and protein ubiquitination [15]. Our recent work has demonstrated that expression of the human mutations CSPα L115R or L116Δ in a neuronal cell line, is associated with a significant elevation of BK channel density at the cell surface. To extend these observations, in the present study we have analyzed human post-mortem ANCL brain specimens by western blot. Expression of the pore-forming BKα subunit in ANCL and Alzheimer’s disease (AD) was compared. Our data demonstrate that BK channel protein expression is higher in human post-mortem ANCL compared with age-matched control specimens. We further show that BKα subunit levels are not altered in brain cortical tissue from AD patients. These results suggest that dysregulation of BKα subunit expression is selective for the pathogenic cascade of events underlying ANCL.

## Results

### BK channel expression is elevated in ANCL

**Fig 1:**
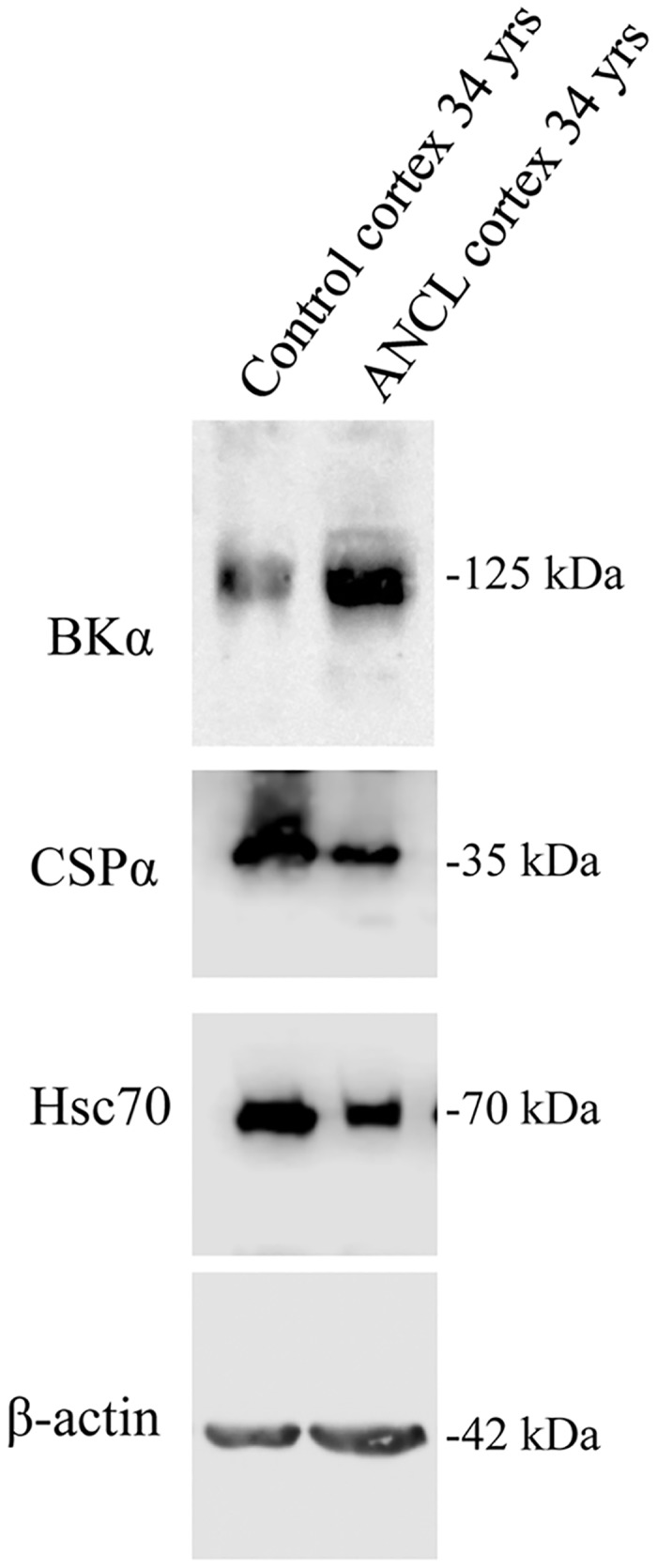
BKα channel expression is elevated in ANCL. Western analysis of BKα channel, CSPα, and Hsc70 detected in 20μg of crude synaptosome fraction prepared from human cortex Control (34 yrs) and ANCL (L 116 Deletion; 36 yrs) as indicated. Detection of β-actin on the same blot was used to verify equal loading between the various lanes.

BKα subunit expression was evaluated in crude synaptosome fractions (P2) prepared from a post-mortem ANCL cortex sample obtained from a 36 year old male with the CSPα mutation L1160Δ and a control cortex sample derived from a 34 yr old male (Fig 1). A higher level of BKα subunit (~2.5 fold increase) was found in ANCL cortex compared with the control sample. No difference was detected in the cellular levels of β-actin. This increase in BK channel expression in human ANCL cortex is consistent with our previous work showing that BKα levels are higher in CSPα KO mice and neuronal cell lines expressing mutated forms of CSPα, including L116Δ [13,14].

**Fig 2:**
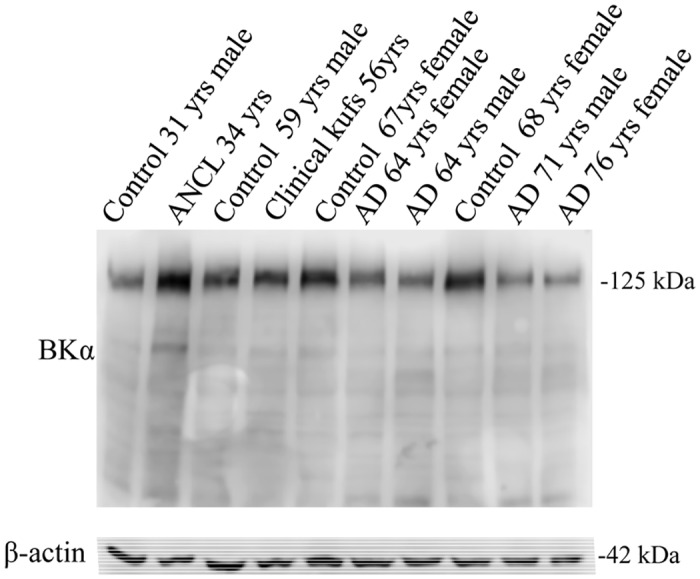
BKα channel expression is elevated in ANCL. Western blot analysis of BKα channel detected in 35 μg of crude synaptosomes prepared from human cortex as indicated. Detection of β-actin on the same blot was used to verify equal loading amongst the various lanes. For the 10 human samples the BKα values were (left to right); 203000, 496000, 336000, 322000, 399000, 256000, 227000, 406000, 215000, 154000.

Neuronal Ceroid Lipofuscinoses (NCLs), also known as Batten Disease, are a group of at least 14 distinct genetic disorders characterized by lysosomal accumulation of auto-fluorescent lipofuscin in neurons and neurodegeneration in the CNS. ANCL is the only NCL type with dominant inheritance. It is a rare, autosomal dominant adult-onset, neurodegenerative disorder, and to date, less than 100 proven isolated or familial cases have been reported. To examine if the CSPα-related changes in synaptic protein machinery are involved in diseases other than ANCL, we investigated the expression of BKα subunit levels in post-mortem Alzheimer’s disease (AD) cortex samples. Fig 2 shows AD cortex samples from a female of 64 years, a male of 64 years, a male of 71 years and a female of 76 years. No change in BKα subunit expression was observed in the four AD cortex specimens evaluated compared with age-matched controls. The detection of β-actin in the same samples is shown for reference. We also examined BKα subunit expression in a post-mortem sample from an individual with clinically suspected ANCL, who was negative for mutations in the gene *DnaJC5*, the gene coding for the CSPα protein. BK channel levels were not altered in this tissue compared with age-matched control. These data suggest that the increase in expression of the BKα channel in post mortem cortex is selectively associated with mutations in the gene *DNAJC5*.

**Fig 3:**
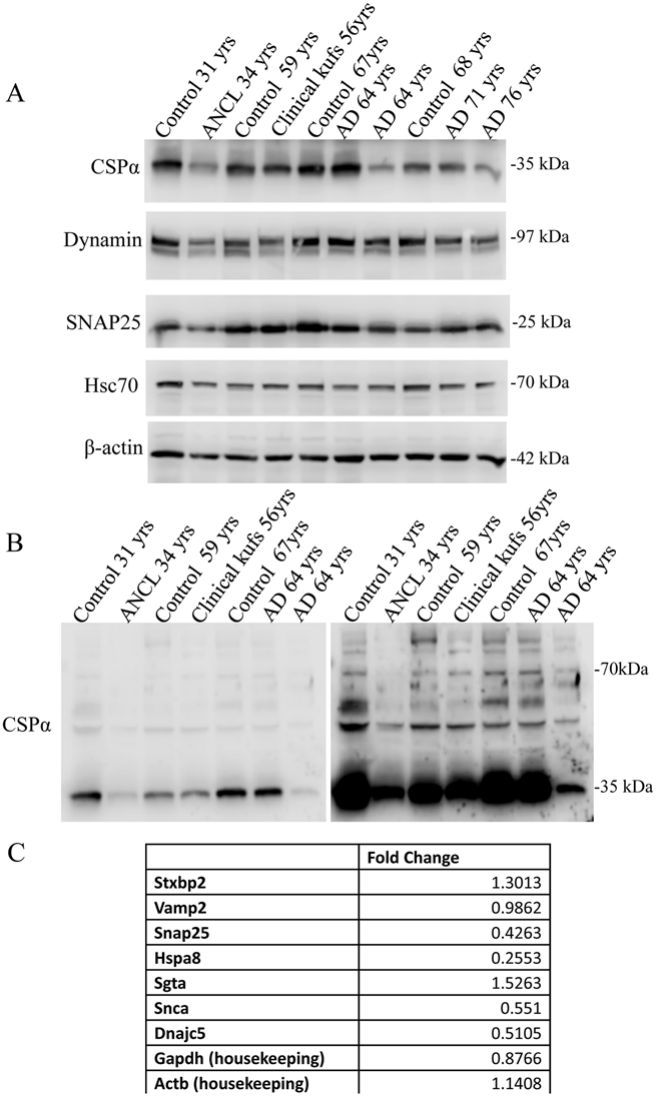
CSPα expression is lower in ANCL and some AD patients. **(A)**Western analysis of CSPα, Dynamin 1, SNAP25, Hsc70 and β-actin detected in synaptosome fractions prepared from human cortex as indicated. For the 10 human samples the CSPα values were 671000, 459000, 583000, 530000, 684000, 690000, 351000, 432000, 397000, 342000. SNAP25 values were 2540000, 1640000, 2880000, 3040000, 3210000, 2530000, 2460000, 2050000, 2430000, 2140000, dynamin values were 3290000, 2160000, 3060000, 2580000, 3410000, 3500000, 3010000, 3240000, 2530000, 2300000 (B) Immunoblot showing CSPα monomer expression (left panel) and longer exposure showing high molecular weight CSPα oligomers (right panel) (C) mRNA levels for the indicated proteins (fold change compared to control brain tissue).

CSPα KO mice appear normal at birth, but around postnatal day 20, they develop progressive neurodegeneration, followed by early lethality between days 40–80. However, CSPα heterozygote mice, which have reduced levels of CSPα, are asymptomatic [6]. The extent to which CSPα expression falls before initiating degenerative consequences is not yet determined, but would appear to be <50% of normal, based on survival data from heterozygous mice. CSPα is a heavily palmitoylated protein that migrates as a monomer of ~35 kDa following separation by SDS-PAGE. In cortical tissue from an ANCL patient with the L116Δ mutation, CSPα levels were reduced compared with an age matched control (Figs 1 and 3), consistent with a report by Noskova et al [2]. Fig 3 shows that SNAP25 and dynamin 1, two proteins in the cellular CSPα pathway, are also reduced in ANCL cortex [16–19]. We also observed that mRNA levels for CSPα and SNAP25 were reduced compared with levels in the control brain tissues. The sample identified as clinically suspected ANCL, but negative for mutations in *DnaJC5*, did not have a similar reduction in CSPα at either the mRNA or protein levels.

CSPα is capable of forming oligomers. We and others have detected 70 kDa detergent-resistant CSPα dimers in rat brain [20] and various cell lines [21,22] and have shown that amino acids between 83 and 136 are important for CSPα self-association [23]. Whether CSPα oligomers display an altered chaperone specificity or chaperone-independent functions is not known. Mutant forms of CSPα (i.e. L115R and L116Δ), exhibit a high potency to oligomerize [5,24]. To what extent ANCL pathology results from a reduction in monomeric CSPα versus the generation/action of CSPα oligomers is not clear. We asked if crude synaptosomal (P2) fractions would contain an abundance of the higher molecular weight CSPα oligomers. To resolve CSPα oligomers by SDS-PAGE, we first solubilized synaptosomes in 0.5% (v/v) Triton-X100/PBS at 4°C prior to incubation in Laemmli sample buffer at 37°C for 1 hour. The results in Fig 3 demonstrate that the crude synaptosome preparations from post-mortem ANCL have reduced levels of both CSPα monomer and oligomers compared with an age-matched control sample.

Fig 3 also shows that CSPα monomer levels were reduced in three of the four AD samples evaluated, relative to the age-matched controls; only the 64 yr old female did not show a reduction in CSPα. There is substantial variability in the extent of reduction in CSPα levels. Notably, CSPα levels were found to be decreased by 35% in the 68yr old control cortex compared with cortex of the 67, 59 and 36 yr controls. Chandra and colleagues have recently reported a decrease in CSPα levels in postmortem AD cortex [16]. Together these observations suggest that CSPα levels are generally reduced in subpopulations of AD patients and that an age-dependent, non-AD dependent reduction in CSPα levels may also occur. In contrast to our data in ANCL tissue and CSPα knockout mice, we did not observe an increase in BKα channels in AD specimens, indicating that a partial reduction in CSPα levels did not result in the increase in BKα subunit levels in AD post-mortem cortex. These findings are consistent with our observations in CSPα heterozygous mice, which have approximately half the normal level of CSPα, but normal BK channel expression and are asymptomatic [6]. Together these findings indicate that the observed increase in BK channels correlates with total CSPα deficiency, as observed in the CSPα knockout mice, or with the heterozygous CSPα mutation L116Δ as seen in ANCL, but not with partial reductions in the expression of normal CSPα protein, as observed in heterozygous CSPα mice.

### Time course of CSPα oligomer expression in CAD cells

**Fig 4:**
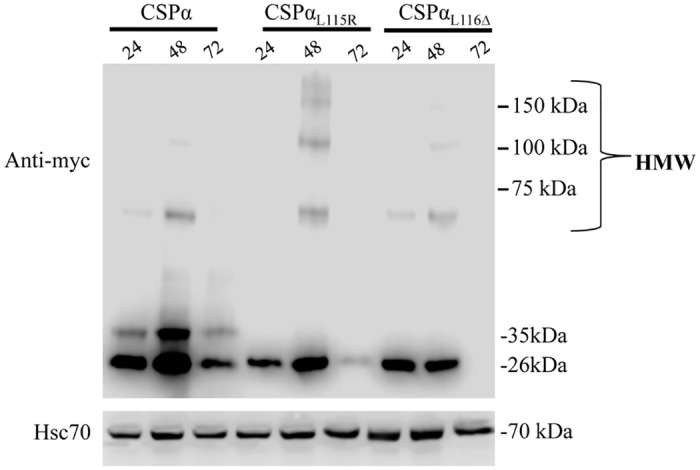
Time course of oligomer expression in CAD cells. Immuoblotting of myc-tagged CSPα, CSPα_L115R_ or CSPα_L1160Δ_ 24, 48 and 72 hrs post-transfection. 30ug of CAD cell lysates were evaluated and endogenous Hsc70 detection is shown for reference. Results are representative of 4 separate experiments.

Three obvious scenarios arise from the fact that CSPα oligomers are not abundant in crude synaptosome fractions (P2) prepared from post-mortem ANCL cortex; (1) CSPα oligomers accumulate during ANCL disease progression, but do not co-fractionate with CSPα monomers anchored to synaptosomes (2) CSPα oligomers are quickly cleared from neurons or (3) CSPα oligomers are more abundant in brain regions other than cortex. We therefore examined the rate of clearance of the high molecular weight CSPα oligomers following transient transfection of the murine CNS-derived catecholamine (CAD) cell line. Fig 4 shows the expression of myc-tagged CSPα, CSPα_L115R_ and CSPα_L116Δ_ at 24 hrs, 48hrs and 72hrs following transfection. As expected, myc-tagged wild type CSPα is expressed as unpalmitoylated (26kDa), a palmitolyated monomer (35kDa) and a dimer (70kDa) species in CAD cells. CSPα_L115R_ and CSPα_L116Δ_ are primarily expressed as the unpalmitoylated 26kDa species and high molecular weight CSPα oligomers. CSPα oligomers do not build up in CAD cells and both monomer and high molecular weight oligomers are significantly cleared three days post transfection. The expression of endogenous Hsc70 is shown for reference. Taken together, while CSPα oligomers have been consistently documented and extensive oligomerization is observed with the CSPα mutants, L115R and L116Δ, the oligomers are not retained for a longer window of time in CAD cells compared with the CSPα monomer (Fig 4) and are not particularly abundant in crude synaptosomal fractions from ANCL cortex (Fig 3).

**Fig 5:**
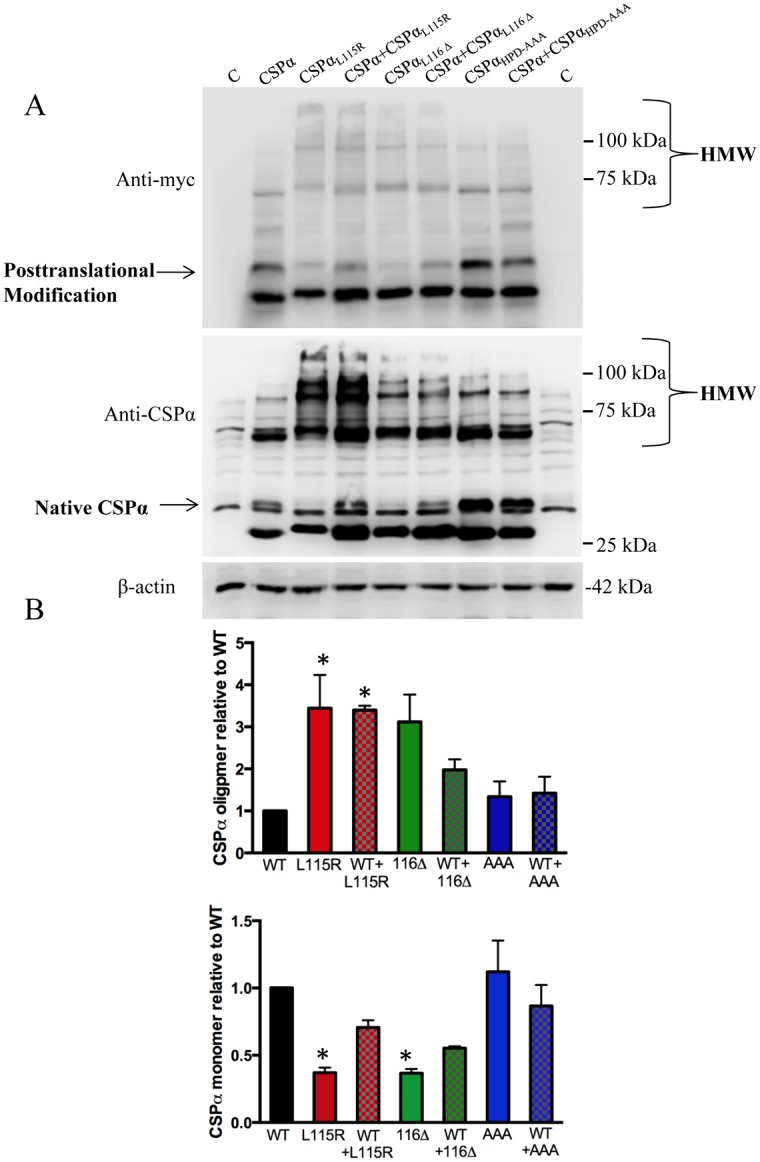
CSPα oligomerization is greater in CSPα_L115R,_ CSPα_Δ116_ mutants. **(A)** CAD cells were transiently transfected with 0.75 μg cDNA encoding myc tagged wild type CSPα or 1.0 μg cDNA encoding either CSPα_L115R_, CSPα_L116Δ_ or CSPα_HPD-AAA_ as indicated and lysed 48 hours post-transfection. CSPα monomer and oligomers were detected in CAD cell lysates by western blot with anti-myc (upper panel) and anti-CSPα (middle panel). Detection of β-actin was used to verify equal loading amongst the various lanes. **(B)** Quantification of CSPα high molecular weight oligomers (starting from 70kDa) CSPα monomers. *p < 0.01. Results are from 4 independent experiments.

We then investigated if mutant CSPα_L115R_ and CSPα_L116Δ_ oligomerized in the presence of wild type CSPα. Fig 5A shows that two days following transfection, CSPα_L115R_ and CSPα_L116Δ_ but not CSPα_HPD-AAA,_ were associated with increased levels of high molecular weight CSPα oligomers both in the absence and presence of wild type CSPα. The 35 kDa myc-CSPα monomer was reduced in the presence of CSPα_L115R_ and CSPα_L116Δ,_ consistent with a recent report showing that wild type and mutant CSPα co-oligomerize, leading to a decrease in functional chaperone activity [24]. Very low levels of the 35kDa CSPα_L115R_ and CSPα_L116Δ_ in CAD cells indicate that these mutants can be posttranslationally modified. These observations were confirmed utilizing an anti-CSPα polyclonal antibody generated to the C terminus of CSPα (Fig 5B). Note that low levels of the 35kDa & 70kDa species of endogenous CSPα are found in CAD cells (lanes 1&9).

**Fig 6:**
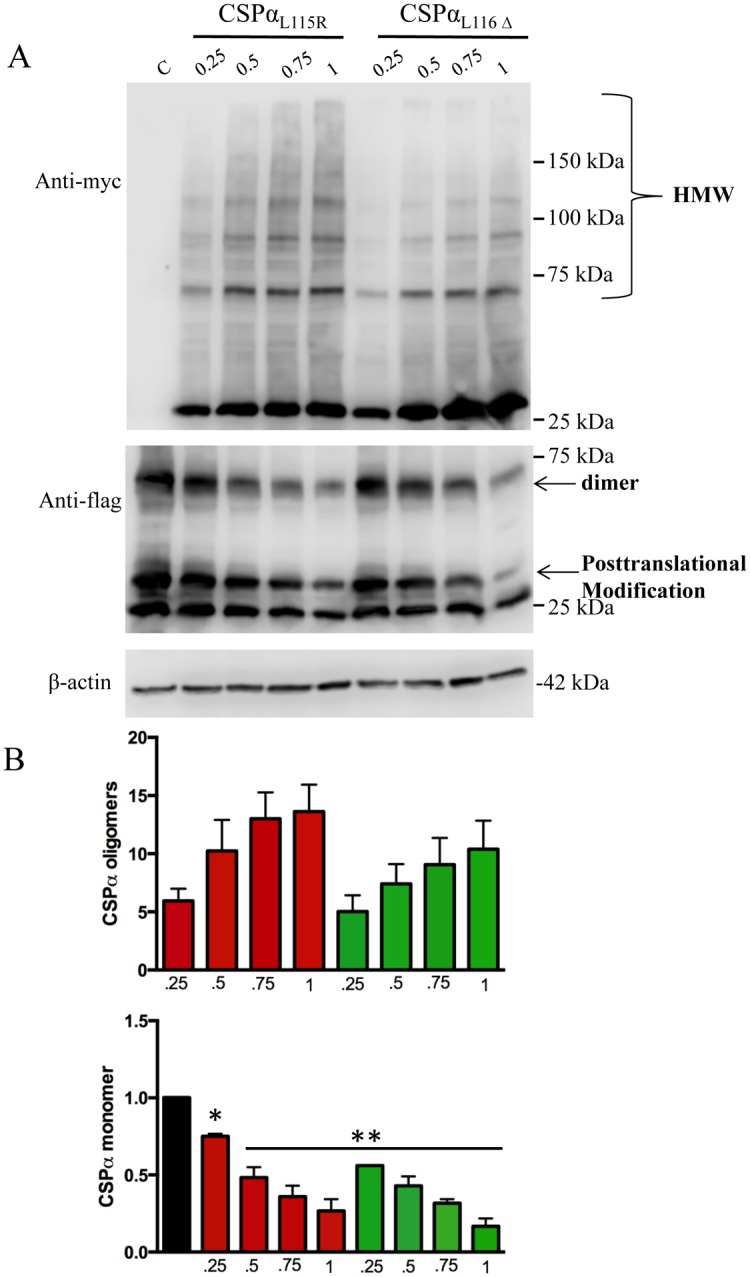
Oligomerization of CSPα_L115R,_ CSPα_Δ116_ mutants is concentration-dependent. CAD cells were transfected with 0.75μg of flag-tagged WT CSPα and 0.25, 0.5, 0.75, 1 μg of either CSPα_L115R_, or CSPα_L116Δ_ as indicated and lysed 48 hours post transfection. Mutant CSPα was detected with anti-myc (upper panel) and WT CSPα was detected with anti-flag (lower panel). Detection of β-actin was used to verify equal loading amongst the various lanes. **(B)** Quantification of CSPα high molecular weight oligomers (starting from 70kDa) and CSPα monomers. Differences between CSPα_L115R_ and CSPα_L116Δ_ oligomers are not significant, *p < 0.02; **p < 0.001. Results are from 3 independent experiments.

Next we ‘titrated’ the effect of CSPα_L115R_ and CSPα_L116Δ_ on FLAG-tagged CSPα. CAD cells were transfected with 0.75μg of flag-tagged CSPα DNA encoding in the presence and absence of 0.25, 0.5, 0.75 and 1μg of DNA encoding myc-tagged CSPα mutants. Fig 6 demonstrates that CSPα oligomers were detected at low (0.25μg) expression of CSPα_L115R_ and CSPα_L116Δ_ and observed to increase at high (1μg) expression of the CSPα mutants. Increasing the proportion of mutant CSPα also decreased the proportion of posttranslationally-modified FLAG-tagged CSPα (35kDa). CSPα_L116Δ_ oligomerization was lower than that found for CSPα_L115R,_ consistent with previous results [24]. Taken together, these data show that increasing the mutant:WT CSPα ratio, correlates with a loss of monomer and an increase in oligomer formation.

### Cellular CSPα-BK channel complexes

**Fig 7:**
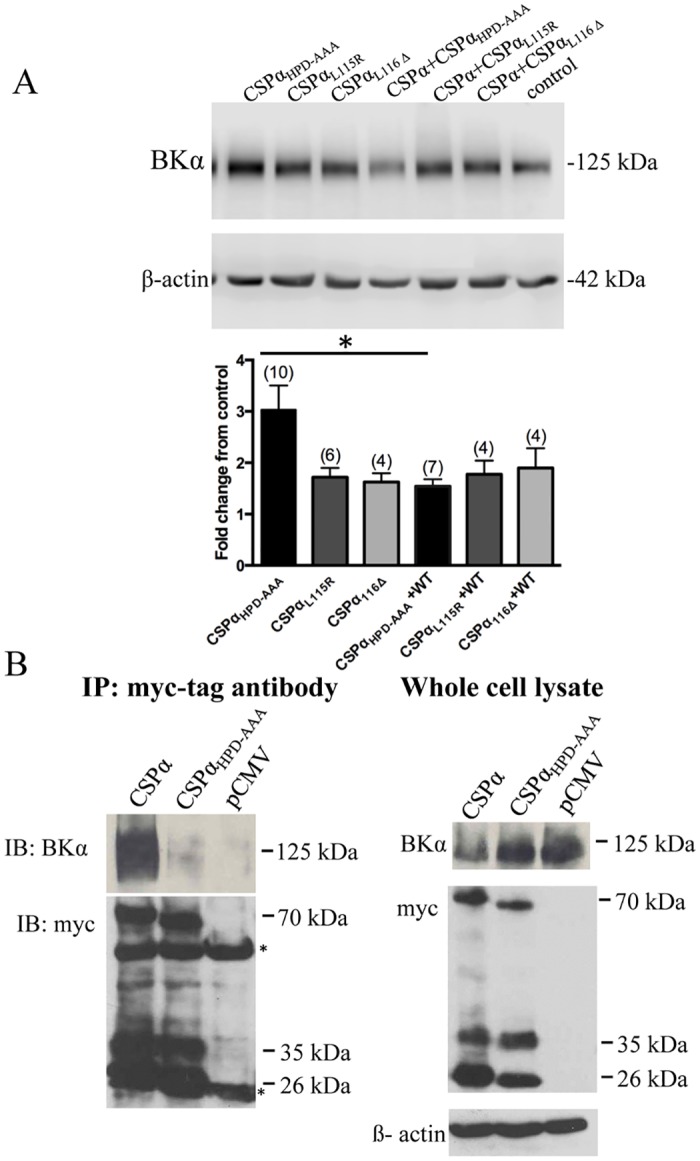
BK channel co-immunoprecipitates with CSPα but not CSPα_HPD-AAA_. **(A)** CAD cells were transiently transfected with 1.0 μg cDNA encoding either CSPα_L115R_, CSPα_L116Δ_ or CSPα_HPD-AAA_ in the presence and absence of 0.75 μg cDNA encoding myc tagged wild type CSPα and lysed 48 hours post-transfection. Western analysis and quantification of BK channel is shown; *p<0.05. Detection of β-actin on the same blot was used to verify equal loading between the various lanes. **(B)** CAD cells were transiently co-transfected with 1 μg BK channel and 0.75 μg myc-tagged CSPα, 0.75 μg myc-tagged CSPα_HPD-AAA_ and 0.75 μg pCMV (negative control). 0.7 mg of soluble cell lysate was subjected to immunoprecipitations with anti-myc monoclonal followed by Western blot analysis with anti-BK channel polyclonal and anti-myc monoclonal. The 55 kDa and 26kDa represent the heavy and light chain of the monoclonal myc-tag antibody. The right panel shows total cellular protein (input). Data are representative of three experiments.

CSPα is a presynaptic synaptic vesicle protein that regulates BK channels, most likely by interacting with presynaptic BK channels. We have previously determined that the mutant CSPα_HPD-AAA_ as well as the human disease-associated CSPα mutations CSPα_L116Δ_ and CSPα_L115R_ are capable of increasing BK channel cell surface expression and current density [13]. While CSPα_L116Δ_ and CSPα_L115R_ increase BK current at the membrane, the increase is not as large as that observed with CSPα_HPD-AAA._ These observations indicate that BK channels are trafficked to the surface and are functional in the presence of mutant CSPα’s. We have also previously demonstrated that when BK channels are expressed at high levels, wild type CSPα reduces BK channel expression in a dose- and time-dependent manner without altering BK β channel subunit expression [14]. Fig 7A shows that WTCSPα limits the CSPα_HPD-AAA_-induced increase in BK channel expression, but does not influence the CSPα_L116Δ_ and CSPα_L115R_-induced increases in BK channel expression. We speculate that the reason why the 0.75:1 ratio of WTCSPα:CSPα_L115R_ or WTCSPα:CSPα_L116Δ_ does not block the increase in BK channel expression may be a consequence of oligomerization of WTCSPα by the CSPα_L115R_ and CSPα_L116Δ_ mutants (Fig 6). Chaperones are known to bind and unbind client proteins with fast kinetics, making the identification of chaperone complexes challenging. Since we were unable to capture stable CSPα-BK channel complexes from wild-type mouse brain using a classic immunoprecipation strategy [13], we investigated the nature of the CSPα-BK channel association in CAD cells co-transfected with BK channel and either myc-tagged CSPα or myc-tagged CSPα_HPD-AAA_. Fig 7B shows that WTCSPα, but not CSPα_HPD-AAA,_ co-immunoprecipitates with BK channels. The pCMV vector control shows that BK channels are not detectable in the immunoprecipitate when CSPα is not co-transfected. Total lysates are shown in the right-hand panel. Taken together, these results indicate that CSPα and BK channel can associate, while this is less likely for CSPα_HPD-AAA_ mutant and BK channels. Such observations support the idea that CSPα_HPD-AAA_ cannot function to limit BK channel expression.

### The J protein network is compromised in ANCL

**Fig 8:**
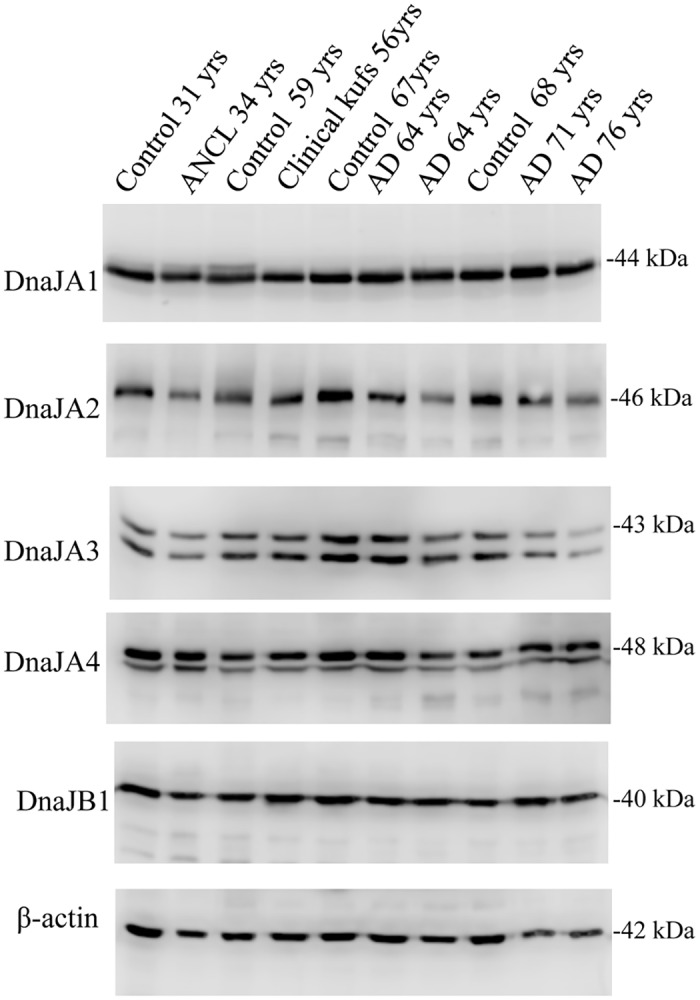
Expression of DnaJA1, DnaJA2, DnaJA3, DnaJA4 and DnaJB1, in human cortex samples. Western analysis of the indicated proteins in synaptosome-enriched fractions prepared from human cortex. For the 10 human samples the DnaJA2 values were: 77500, 57900, 85900, 78500, 98100, 69700, 52500, 83800, 64300, 56400. The DnaJ3A values were: 298000, 224000, 356000, 416000, 554000, 509000, 375000, 342000, 234000, 181000.

Finally, we asked if the expression of select J proteins is altered in crude synaptosomes from post-mortem ANCL cortex. CSPα is a member of a large J protein family that is central to cellular protein homeostasis pathways [25,26]. While pathological neurodegeneration in ANCL is directly correlated with mutations in CSPα, the extensive synapse dysfunction associated with disease progression may be expected to compromise other members of the chaperone network. Exhaustion of molecular chaperones would render many cellular pathways vulnerable, triggering a generalized collapse of proteostasis. Reductions were found in DnaJA2 (26%) and DnaJA3 (25%) levels in post-mortem ANCL cortex synaptosomes (Fig 8). These observations suggest that disease progression in ANCL may eventually lead to an impairment of the J protein network.

## Discussion

ANCL is a rare neurodegenerative disease caused by L115R and L116Δ mutations in the synaptic vesicle protein CSPα. The pathogenesis of ANCL is not established. In this study we investigated BKα channel expression in ANCL post-mortem cortex with a L116Δ mutation. Our previous work demonstrated that the presynaptic chaperone CSPα limits BK channel density and that ectopic expression of CSPα_L115R_ and CSPα_L116Δ_ results in elevation of BK channel expression in neuronal cell culture models [13,14]. We have also shown that CSPα KO mice have a 2.5 fold increase in BK channel levels in the brain [13]. Here we show that BKα subunit levels are elevated in crude synaptosomes from human ANCL cortex. Thus, we conclude that the pathological sequence of events in ANCL involves elevations in BK channel levels, which may contribute to the dysregulation of neuronal excitability.

The neural chaperone network that maintains the balance between protecting functional proteins and preventing accumulation of mis-folded proteins is elaborate. CSPα is a pivotal element of the presynaptic proteostasis machinery [6]. Biochemical analysis of CSPα KO mice has identified SNAP25 and dynamin1, proteins critical to synaptic vesicle recycling, as CSPα-protein clients [16–19,27]. The decrease in SNAP25 and dynamin1 levels in CSPα KO mice suggest that these client proteins are degraded rather than refolded and retained in this model [27]. In contrast, BK channel levels increase in CSPα-KO mice, suggesting that either delivery of the channel to the synapse is increased or removal of the channel is decreased implicating BK channel proteostasis in the cellular pathway of CSPα-mediated neuroprotection. Other proteins that interact with CSPα and might be involved in CSPα’s neuroprotective activity have been identified, including voltage dependent Ca^2+^ channels, Gα_s_, syntaxin, rab3, synaptotagmin [28–37].

The cysteine string region is a heavily palmitoylated region that anchors the chaperone, CSPα, to synaptic vesicles [38]. Mutations of CSPα, L115R and L116Δ, have been shown to interfere with palmitoylation and to promote a high potency to oligomerize [5,24]. CSPα oligomers with and without palmitoyl groups have been identified [5,24]. In contrast to the CSPα monomer, high molecular weight CSPα oligomers do not activate Hsc70 ATPase for conformational work [24]. Protein oligomerization and buildup of protein aggregates is a common event in several neurodegenerative diseases, nonetheless, we were unable to detect an over-abundance of oligomers. In fact, both monomeric and oligomeric species of CSPα are reduced in ANCL synaptosomes. In cell culture, CSPα oligomers were reduced 3 days following transfection. This temporal pattern is similar to the expression of ectopically expressed CSPα monomer and thus it appears that CSPα oligomers do not progressively build up. Recent reports demonstrate that oligomers are poly-ubiquitinated [24] consistent with our observations that neurons effectively clear CSPα oligomers. Furthermore, in CAD neuroblastoma cells, wild type CSPα reverses the CSPα_HPD-AAA_-induced increase but not the CSPα_L116Δ_ and CSPα_L115R_- induced increase in BK channel levels. These observations indicate that at the ratio of 0.75WT:1mutant; mutations in the J-domain (i.e. CSPα_HPD-AAA_) but not mutations in the cysteine string region (i.e. CSPα_L116Δ_ or CSPα_L115R_) are compensated.

A diverse number of human neurodegenerative disorders are caused by mutations in members of the J protein family DnaJC29 in addition to DnaJC5 (CSPα), for example DnaJB2, DnaJC6, DnaJC13, DnaJC19 [39,40]. Loss of co-chaperone activity and/or gain of additional modes of chaperone activity almost certainly underlies the pathophysiology of these different diseases. Additionally, the buildup of misfolded proteins in common neurodegenerative diseases, such as Alzheimer’s disease and Parkinson’s disease implies that chaperone activity is compromised, however a comprehensive understanding of the identity of the specific defective chaperone(s) in these diseases is not clearly established. It is clear that CSPα confers synapse protection [6] and that synaptic function is compromised in the absence of CSPα [6,17–19,27,41]. While partial reductions in CSPα (e.g. CSPα heterozygote mice) are not pathogenic, the reduction in CSPα monomer together with the assembly of CSPα into oligomers (e.g. ANCL), does result in neurodegeneration.

In conclusion, we provide evidence that the L116Δ mutation of the presynaptic chaperone CSPα, increases BKα subunit expression in crude synaptosomes from post-mortem cortex. Cell surface expression of BK channels is subject to elaborate regulatory mechanisms. BK channels are positioned to influence synaptic transmission and excitability. Our work points to the dysregulation of BK channels in ANCL. Together, these data suggest that while reinforcement of CSPα co-chaperone activity may prove effective therapy for ANCL, effectively overcoming the oligomerizing and sequestering activity of wild type CSPα by the human CSPα mutations remains an important consideration.

## Materials and Methods

### Preparation of fractions from human cortex

This study of the brain tissues was conducted according to protocols approved by the Institutional Review boards of the New York State Institute for Basic Research in Developmental Disabilities and the Institutional Review Board of Massachusetts General Hospital. The brain tissue with DNAJC5 deletion was from an individual that was previously published [42]. The specimen from individual with ANCL negative for DNAJC5 mutation was obtained from the Human Brain and Spinal Fluid resource Center in Los Angeles, CA. The rest of the tissues were obtained from the Brain Bank for Developmental Disability and Aging at the New York State Institute for Basic Research in Developmental Disabilities. For all tissues written consent for research participation was obtained from the affected individual or from next of kin. The study was conducted according to principles of the Declaration of Helsinki.

Briefly, frozen human cortex samples were homogenized in 0.7 mls of ice cold 0.32M sucrose, 10mM HEPES, 1 mM EGTA, 0.1 mM EDTA and 0.3 mM PMSF with 20 up and down strokes using a plastic mini homogenizer. The homogenate was centrifuged at 4°C for 5 min at 700 x g and the supernatant (S1) collected. The S1 supernatant was then spun for 15 min at 22,000 x g and the resulting supernatant (S2) was discarded. The pellet (P2) was washed by re-suspension buffer and then re-centrifuged at 22,000 x g. The final pellet, representing washed-crude synaptosomes, was re-suspended in 0.4 ml of buffer.

### Cell culture

CAD (CNS catecholaminergic derived) mouse neuroblastoma cells stably expressing BKα subunit were seeded into 6 well plates and grown in DMEM/F12 medium supplemented with 10% fetal bovine serum, 1% penicillin/streptomycin and 0.5mg/ml zeocin, as previously described [13]. For transient transfection, CAD cells were washed in PBS and transiently transfected using with the indicated amount of cDNA and 6 μl of Lipofectamine-2000 (Invitrogen) per dish. Reagents were mixed in 0.2 ml of Opti-MEM medium and then diluted to a total volume of 1 ml with DMEM. After 6 hours the medium was replaced with DMEM/F12 supplemented with 1% fetal bovine serum, 1% penicillin/streptomycin and 0.5mg/ml zeocin. Cells were lysed in 40 mM Tris (pH 7.4), 150 mM NaCl, 2 mM EDTA, 1 mM EGTA, 1 mM Na_3_ VO_4_, 0.1% SDS, 1% (v/v) Triton X-100, 0.5 mM PMSF and protease inhibitor (Sigma) at 4°C for 1 hour. Lysates were centrifuged at 10000 x g for 5 minutes at 4°C and the supernatant (soluble fraction) was collected and stored at -70°C. Protein concentration of the soluble CAD cell lysate was determined using the Pierce BCA protein assay.

### Immunoblotting

Proteins were separated by SDS-PAGE and electrotransferred from polyacrylamide gels to nitrocellulose membrane (0.2 μm pore size). Membranes were blocked in tris-buffered saline (TBS) containing 0.1% Tween 20, 1% BSA and then incubated with primary antibody overnight at 4°C. The membranes were washed and incubated with horseradish peroxidase-coupled secondary antibody for ~2 h at room temperature. Bound antibodies on the membranes were detected by incubation with the LiCor WesternSure chemiluminescence reagent (Mandel) and exposure to Cdigit, LiCor. The chemiluminscent signals were quantified using image studio digits software (Mandel). Primary antibodies were obtained as follows: BK monoclonal, c-myc monoclonal, flag monoclonal and dynamin monoclonal (BD Biosciences). SNAP25 monoclonal (Sternberger monoclonals). Syntaxin monoclonal, Hsc70 monoclonal, and β-actin monoclonal (Sigma-Aldrich). DnaJA2 monoclonal was from US Biologicals, DnaJA1 monoclonal and DnaJA4 monoclonal was from Abnova. DnaJB1 polyclonal was from Enzo Life Sciences. DnaJA3 monoclonal was from Pierce, Thermo scientific. Anti-DnaJC5 polyclonal was prepared as previously described [20].

### Quantitative PCR

Total RNA was isolated from frozen brain tissues using Trizol reagent (Life technologies) and RNeasy kit (Qiagen) according to the manufacturer’s protocol. Quantitive real-time RT-PCR was done using custom RT^2^ profiler PCR Array Format A (Qiagen) according to the manufacturer’s protocol. Data analysis was done using ΔΔCt method and automated software from Qiagen.

### Co-Immunoprecipitation

CAD cells were transiently transfected with cDNA and 24 hrs later were lysed in 1 ml 1% v/v Triton X-100 in PBS and protease inhibitor (complete, EDTA-free, Sigma). Lysates were centrifuged at 15,000 rpm for 15 min at 4°C, the supernatant collected and precleared with Protein A/G-coupled agarose beads (50% w/v slurry). 5μg of anti-myc monoclonal was incubated overnight at 4°C with the precleared lysates. Following the addition of 20μl of Protein A/G coupled beads (50% slurry) and a subsequent 2 hr incubation at 4°C, the lysates were centrifuged at 5,000 rpm for 2 min. Pellets were washed thrice with 0.1% v/v Triton X-100 in PBS. Proteins were eluted from agarose beads with 2X Laemmli sample buffer and separated on SDS-PAGE.

### Statistics

All values are presented as the mean ±SEM. Calculations were performed using GraphPad Prism 6 software.
